# Lemmel Syndrome Without Choledocholithiasis as a Cause of Recurrent Hepatocellular Injury: A Case Report

**DOI:** 10.7759/cureus.96929

**Published:** 2025-11-15

**Authors:** Kyoko Hanaoka, Shingo Shimada, Yoichi Miyaoka, Yuya Kanemitsu, Nobuhiko Abe

**Affiliations:** 1 Internal Medicine, Abashiri-Kosei General Hospital, Abashiri, JPN; 2 Surgery, Otaru General Hospital, Otaru, JPN; 3 General Surgery, Abashiri-Kosei General Hospital, Abashiri, JPN

**Keywords:** endoscopic retrograde cholangiopancreatography (ercp), ercp, lemmel syndrome, obstructive jaundice, periampullary duodenal diverticula, transaminitis

## Abstract

Lemmel syndrome is a rare cause of stone-negative biliary obstruction due to external compression of the distal common bile duct by a periampullary duodenal diverticulum (PAD). We report a 67-year-old man with recurrent episodes of liver dysfunction due to Lemmel syndrome. On admission, laboratory tests showed marked hepatocellular injury with cholestasis. Contrast-enhanced CT demonstrated a PAD abutting and compressing the distal bile duct with upstream dilatation, without choledocholithiasis. Endoscopic retrograde cholangiopancreatography (ERCP) with sphincterotomy and balloon sweeping yielded sludge-like bile, after which liver tests rapidly improved. This case underscores the need to consider PAD-related transient obstruction in stone-negative, recurrent hepatocellular flares and highlights the diagnostic-therapeutic value of timely endoscopic biliary decompression.

## Introduction

Duodenal diverticula, including the periampullary duodenal diverticulum (PAD), are relatively common anatomical findings, with a prevalence ranging from 5% to 33% in adults, showing a higher incidence in women and increasing with age [1]. Most cases are asymptomatic and discovered incidentally. However, when a duodenal diverticulum arises 2-3 cm from the ampulla of Vater, it can externally compress the common bile duct, resulting in obstructive jaundice or pancreatitis in the absence of stones or tumors. This condition, first described by Lemmel in 1934, is referred to as "Lemmel syndrome" [2].

Although often overlooked, PAD should be considered in the differential diagnosis of recurrent or unexplained hepatobiliary dysfunction. Here, we report a case of a 67-year-old man who experienced multiple admissions for unexplained hepatocellular injury, ultimately diagnosed as Lemmel syndrome caused by a PAD.

## Case presentation

**Figure 1:**
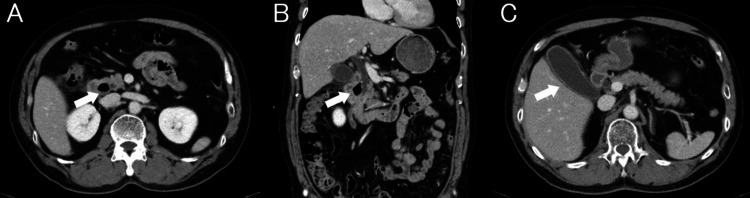
Contrast-enhanced abdominal CT performed during previous hospitalization for liver injury. (A) Axial and (B) coronal reformatted images demonstrate an air-filled periampullary duodenal diverticulum (white arrows) abutting the ampulla and compressing the distal common bile duct. The upstream extrahepatic bile duct is dilated. No choledocholithiasis is identified in the bile duct. (C) The axial image shows no gallstones within the gallbladder (white arrow).

A 67-year-old man with a history of hypertension and heavy alcohol consumption presented with recurrent episodes of liver dysfunction over several years. He had been admitted to other hospitals four times over the past five years for similar episodes characterized by epigastric pain and generalized pruritus, followed by hepatocellular dysfunction that spontaneously resolved within several days, leading to discharge. During these previous admissions, repeated evaluations, including serologic testing for hepatitis B and C viruses and autoimmune markers, revealed no specific etiology. Because the initial liver injury occurred immediately after switching to a generic formulation of allopurinol, drug-induced liver injury due to allopurinol was suspected. However, a drug lymphocyte stimulation test for allopurinol was negative. Biliary dilatation was observed, but no gallstones were detected in previous imaging studies (Figure 1).

During the present admission, he presented with epigastric pain and jaundice. There was no history of overseas travel or consumption of raw meat in the past few weeks, and no new medications had been introduced. Laboratory tests revealed severely elevated transaminase and gamma-glutamyl transferase, hyperbilirubinemia (Table 1).

**Table 1:** Laboratory findings on admission On admission, laboratory data revealed marked elevation of AST and ALT, consistent with acute hepatocellular injury, along with hyperbilirubinemia and a significant rise in GGT, suggesting obstructive jaundice. CRP was elevated, indicating an associated inflammatory process. WBC: white blood cell count; RBC: red blood cell count; Hb: hemoglobin; Ht: hematocrit; PLT: platelet count; AST: aspartate aminotransferase; ALT: alanine aminotransferase; ALP: alkaline phosphatase; GGT: gamma-glutamyl transferase; LDH: lactate dehydrogenase; TP: total protein; Alb: albumin; CRP: C-reactive protein

Parameter	Result	Reference Range
WBC (/µL)	8,600	3,600–9,200
RBC (×10^6/µL)	4.74	4.35–5.55
Hb (g/dL)	14.6	13.7–16.8
Ht (%)	43.2	40.7–50.1
PLT (×10^4/µL)	27.6	15.8–34.8
AST (U/L)	981	13–30
ALT (U/L)	1,304	10–42
ALP (U/L)	158	106–322
GGT (U/L)	821	13–64
LDH (U/L)	801	124–222
Total bilirubin (mg/dL)	5	0.4–1.5
Direct bilirubin (mg/dL)	3.2	0.0–0.3
TP (g/dL)	7.7	6.6–8.1
Alb (g/dL)	4.4	4.1–5.1
CRP (mg/dL)	7.65	<0.3

**Figure 2:**
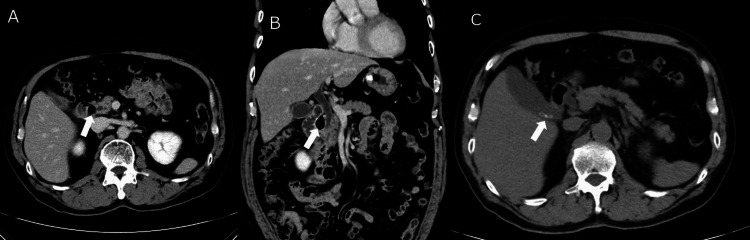
Contrast-enhanced abdominal CT on admission (A) Axial and (B) coronal reformatted images demonstrate an air-filled periampullary duodenal diverticulum (white arrows) abutting the ampulla and compressing the distal common bile duct. The upstream extrahepatic bile duct is dilated. No choledocholithiasis is identified in the bile duct. (C) The axial image shows multiple 3-mm gallstones (white arrow) within the gallbladder.

Contrast-enhanced abdominal CT revealed a markedly dilated common bile duct and the presence of air within a PAD compressing the distal bile duct. Some 3-mm gallstones were identified within the gallbladder, but no stones were observed in the bile duct (Figure 2).

**Figure 3:**
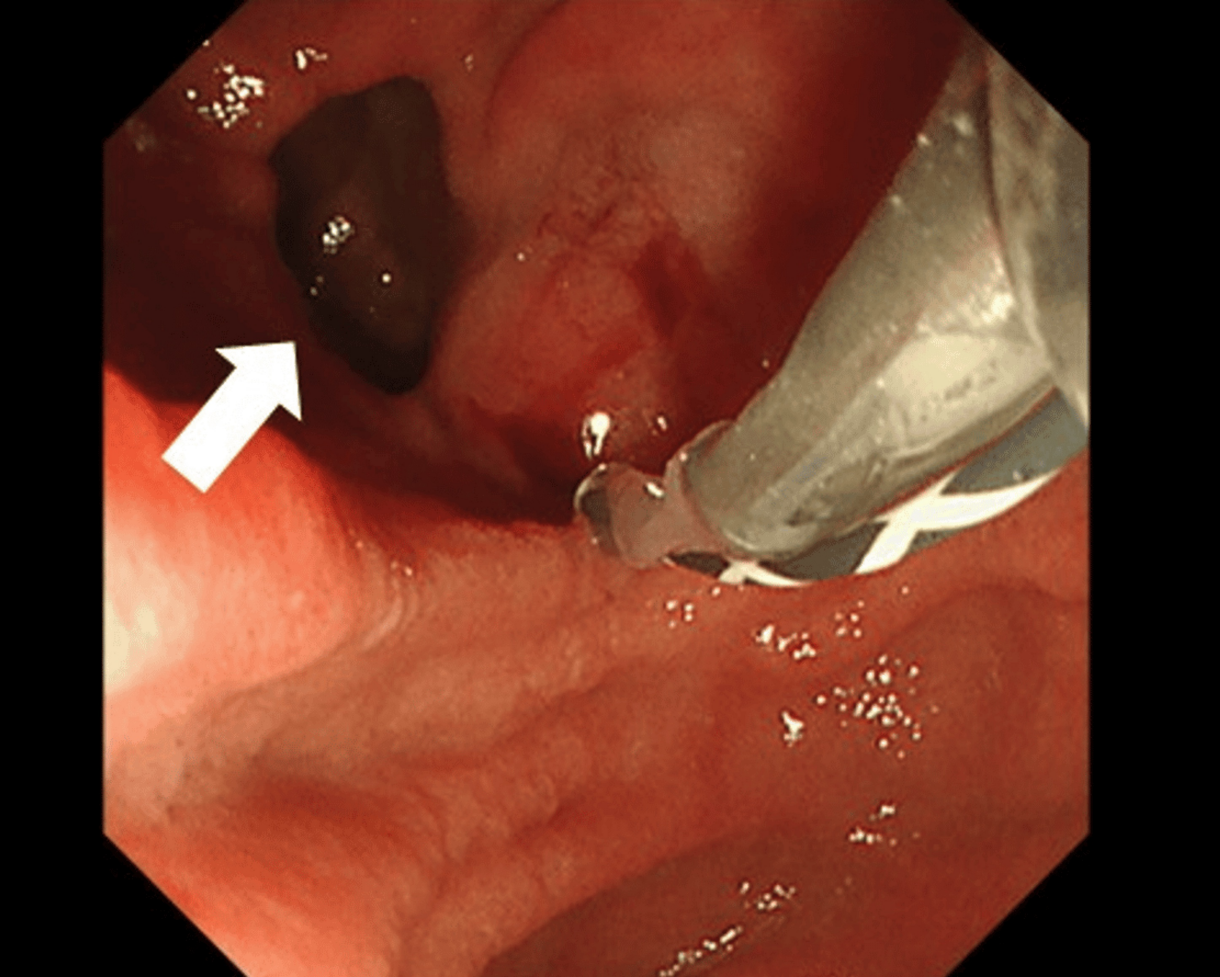
Endoscopic view during the ERCP A periampullary duodenal diverticulum is seen adjacent to the major papilla (white arrow). ERCP: endoscopic retrograde cholangiopancreatography

Endoscopic ultrasound (EUS) and endoscopic retrograde cholangiopancreatography (ERCP) were performed for both diagnostic and therapeutic purposes. ERCP revealed sludge-like bile within the bile duct but no visible stones. Sphincterotomy and balloon sweeping were carried out, which removed the sludge-like material and improved biliary drainage (Figure 3).

**Figure 4:**
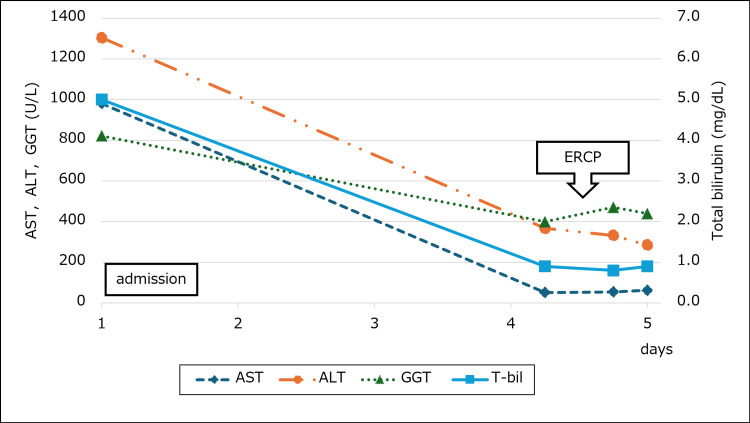
Trends in liver function tests after admission Levels of AST, ALT, GGT, and total bilirubin gradually decreased following admission and improved after ERCP. AST: aspartate aminotransferase; ALT: alanine aminotransferase; GGT: gamma-glutamyl transferase; ERCP: endoscopic retrograde cholangiopancreatography

Before the procedure, the patient’s liver function tests had already shown gradual improvement, and his symptoms, including jaundice and abdominal pain, were alleviating. After ERCP, both laboratory values and clinical symptoms continued to improve (Figure 4).

On hospital day 7, the patient was discharged in good condition under outpatient follow-up.

## Discussion

A PAD is frequently incidental yet clinically important when it compresses or distorts the papilla, producing intermittent, stone-negative outflow obstruction characteristic of Lemmel syndrome. The prevalence of PAD in ERCP cohorts ranges widely (≈5%-33%) and rises with age, underscoring its frequent occurrence during endoscopic procedures [1,2]. Diagnostic delay is common because symptoms and biochemistry fluctuate, and stone-negative imaging may bias clinicians away from a biliary cause. Practical confirmation requires demonstrating a PAD abutting the papilla with ductal dilatation on cross-sectional imaging (CT or magnetic resonance cholangiopancreatography (MRCP)) and, when necessary, endoscopic correlation [3,4].

The proposed mechanism involves external papillary compression by a PAD, which leads to chronic cholestasis. Biliary sludge and stones may subsequently form due to this cholestasis and associated bacterial contamination of the bile. Although transabdominal ultrasonography and CT are valuable for the detection of sludge and stones, they possess certain limitations [5-7]. This paradigm neatly explains our patient’s course: CT showed a dilated common bile duct and diverticular air but no stones, while ERCP documented sludge efflux after sphincterotomy/sweeping, supporting PAD-related stasis as the proximate cause of transient obstruction and relapse. The dramatic yet rapidly reversible aspartate aminotransferase/alanine aminotransferase surges (>1,000 IU/L) are consistent with data showing that severe hepatocellular elevations occur not uncommonly in choledocholithiasis and transient biliary obstruction and promptly improve once the biliary obstruction is relieved [8,9].

The reason for the repeated relapse and remission in our case remains uncertain. However, a recent report described recurrent exacerbations of Lemmel syndrome precipitated by fatty food intake, suggesting that dietary factors may transiently enhance diverticular compression and cholestasis [10]. Such mechanisms might have played a role in the intermittent obstruction observed in our patient.

From a therapeutic standpoint, PAD has historically been associated with more difficult cannulation and an increased risk of adverse events, especially perforation during ERCP, likely related to the absence of a normal muscular layer in the diverticular wall. However, recent evidence suggests that the presence of PAD is no longer regarded as a major obstacle to cannulation. The impact largely depends on the type: PAD can be classified into two types according to papillary location, with the papilla located outside or at the margin of the diverticulum, or the papilla located within the diverticulum. Cannulation tends to be more difficult when the papilla is intradiverticular. Several techniques may be employed to overcome these challenges and reduce the risk of complications. The double-guidewire technique is frequently recommended. When the papilla is intradiverticular, the diverticulum should be everted to allow full exposure of the papilla, and the indication for mechanical lithotripsy should be individualized with careful consideration of the local anatomy if a large common bile duct stone is present. In addition, endoscopic retrograde biliary drainage can be performed initially, followed by ERCP for stone extraction in a staged approach. Applying these strategies enables safe and effective diagnosis and therapy within a single clinical course [1,11,12].

Clinically, when viral, autoimmune, and drug-induced etiologies are excluded in cases of recurrent hepatocellular injury or obstructive jaundice, previously reported cases have identified Lemmel syndrome as one of the underlying causes. Especially in the absence of biliary stones or tumors, Lemmel syndrome should be recognized as an important differential diagnosis. In such situations, clinicians should actively search for a PAD on CT/MRCP and perform early EUS/ERCP, both diagnostically and therapeutically, to achieve prompt biliary decompression even in stone-negative presentations and prevent recurrent admissions [3,4,13].

## Conclusions

Lemmel syndrome should be considered in recurrent, stone-negative hepatocellular flares. PAD can, through mechanisms such as sludge or stone formation, lead to transient common bile duct obstruction and marked transaminase spikes. Targeted imaging and timely ERCP can both confirm the diagnosis and reverse injury.
